# Unraveling Metabolic Changes following Stroke: Insights from a Urinary Metabolomics Analysis

**DOI:** 10.3390/metabo14030145

**Published:** 2024-02-28

**Authors:** Jamie N. Petersson, Elani A. Bykowski, Chelsea Ekstrand, Sean P. Dukelow, Chester Ho, Chantel T. Debert, Tony Montina, Gerlinde A. S. Metz

**Affiliations:** 1 Canadian Centre for Behavioural Neuroscience, Department of Neuroscience, University of Lethbridge, Lethbridge, AB T1K 3M4, Canada; jamie.petersson@uleth.ca (J.N.P.); elani@ualberta.ca (E.A.B.); chelsea.ekstrand@uleth.ca (C.E.); 2 Southern Alberta Genome Sciences Centre, University of Lethbridge, Lethbridge, AB T1K 3M4, Canada; 3 Department of Chemistry and Biochemistry, University of Lethbridge, Lethbridge, AB T1K 3M4, Canada; 4 Department of Clinical Neurosciences, Cumming School of Medicine, University of Calgary, Calgary, AB T2N 1N4, Canada; sean.dukelow@albertahealthservices.ca; 5 Hotchkiss Brain Institute, University of Calgary, Calgary, AB T2N 1N4, Canada; 6 Division of Physical Medicine and Rehabilitation, University of Alberta, Edmonton, AB T6G 2R7, Canada; chester.ho@albertahealthservices.ca

**Keywords:** metabolomics, urine, stroke, nuclear magnetic resonance (NMR) spectroscopy, neurorehabilitation, functional recovery, biomarkers, therapy

## Abstract

The neuropathological sequelae of stroke and subsequent recovery are incompletely understood. Here, we investigated the metabolic dynamics following stroke to advance the understanding of the pathophysiological mechanisms orchestrating stroke recovery. Using a nuclear magnetic resonance (NMR)-driven metabolomic profiling approach for urine samples obtained from a clinical group, the objective of this research was to (1) identify novel biomarkers indicative of severity and recovery following stroke, and (2) uncover the biochemical pathways underlying repair and functional recovery after stroke. Urine samples and clinical stroke assessments were collected during the acute (2–11 days) and chronic phases (6 months) of stroke. Using a 700 MHz ^1^H NMR spectrometer, metabolomic profiles were acquired followed by a combination of univariate and multivariate statistical analyses, along with biological pathway analysis and clinical correlations. The results revealed changes in phenylalanine, tyrosine, tryptophan, purine, and glycerophospholipid biosynthesis and metabolism during stroke recovery. Pseudouridine was associated with a change in post-stroke motor recovery. Thus, NMR-based metabolomics is able to provide novel insights into post-stroke cellular functions and establish a foundational framework for future investigations to develop targeted therapeutic interventions, advance stroke diagnosis and management, and enhance overall quality of life for individuals with stroke.

## 1. Introduction

Stroke is a serious cerebrovascular disease that often leads to permanent motor, sensory, and cognitive impairments. Worldwide, stroke is a major contributor to long-term disability and is ranked as the second leading cause of death [1]. Stroke can be broadly classified into two categories, ischemic and hemorrhagic. Both types of stroke result in cell death which usually spreads to other areas around the primary lesion site [2]. The early identification of stroke type is crucial for timely treatment and, therefore, minimizing brain damage. Imaging techniques such as computed tomography (CT) and magnetic resonance imaging (MRI) are used to confirm the incidence of stroke. Both techniques have limitations, including their high cost and limited accessibility [2,3]. While early endovascular therapy and thrombolytic treatments are useful for damage limitation in the hyperacute phase, rehabilitation is the primary approach to promote the recovery of remaining disabilities. The treatment outcomes, however, can vary greatly between individuals due to the heterogeneity of stroke lesion sites, their extent, and the functional consequences [4]. In order to reduce the risk of suffering from preventable post-stroke disabilities, it is imperative to use a personalized treatment plan that is effective for the prevention and long-term recovery of each patient. One promising technique that has potential to aid in diagnosis and treatment monitoring is the use of fluid biomarkers determined via metabolomics.

Metabolomics, the study of metabolic products or metabolites in living systems, offers a snapshot into the dynamic cellular processes of organs and the overall health of an individual [5]. Additionally, since metabolism is perturbed by abnormal cellular processes, metabolomics provides an integrative view of biochemical processes occurring in an organism [6]. Because several metabolites traverse the blood–brain barrier, biofluid analysis, such as blood and urine, may provide insight into metabolic perturbations in the brain [7], making metabolomics a powerful tool for detecting and monitoring neuropathophysiological states of cerebrovascular diseases. Urine is regularly utilized as a biofluid for studying metabolomic changes because it is non-invasive, easy to collect, and requires minimal sample preparation [8].

Nuclear magnetic resonance (NMR) spectroscopy quantifies metabolites by detecting the unique biochemical signature of metabolites in a biological sample. The NMR spectrum of a biological sample is therefore a composite of the fingerprints of all the molecules present in the sample. NMR is highly reproducible, non-destructive to the sample, and non-biased, it requires little sample preparation and analysis [6,9,10], and it can simultaneously identify metabolites involved in biological processes [5]; however, NMR is only able to quantify metabolites at micromolar concentrations and requires more sample volume, while mass spectrometry-based metabolomic methods are capable of quantifying at the nanomolar level and require much less sample volume. Previously, NMR spectroscopy has been used in the development of biomarkers for stroke [7], Alzheimer’s disease [11], spinal cord injury [12,13], traumatic brain injury [14], and sport-related concussion [15,16]. 

Metabolomic biomarkers are critical for timely diagnosis. Biomarkers can serve as robust indicators of disease state, recovery, or predicted clinical course [17]. Biomarkers, which are indicators of physiological processes and conditions, offer the ability to distinguish between normal and pathological states, evaluate therapeutic efficacy, and prognosticate future disease outcomes [18]. Given the time-sensitive nature of stroke therapies, the development of a rapid and accurate biomarker could improve outcomes significantly. Imaging techniques are not always readily available, can sometimes provide a false negative diagnosis, and are not able to provide an accurate prognosis or advise a patient’s therapy. The diagnostic value of NMR-based metabolomics lies in its translatability into commercial assays, in which biomarkers can be rapidly tested [19]. While NMR, like CT and MRI, comes with a high cost and limited accessibility, its effectiveness in identifying biomarkers is noteworthy. This is due to NMR’s capability to identify a wide range of metabolites in biological samples, which can be readily applied in clinical settings. More importantly, this technique is useful for developing biomarkers to predict and monitor recovery and after the acute phase, which is needed in the field. Thus, it is important to develop alternative techniques that can confirm stroke diagnosis [2], monitor patients through their treatment, and evaluate the effectiveness of treatment programs in precision medicine approaches [20].

A biomarker panel for stroke may elucidate the underlying cellular mechanisms contributing to individual variability in symptom burden, prognosis, and recovery [21,22], thus also revealing potential therapeutic targets. Studies have previously investigated biomarkers for stroke diagnosis [23], prognosis [24], and acute vs. chronic stage [25]. To our knowledge, the current investigation represents the first attempt to identify NMR-based urinary metabolomic biomarkers that could serve as indicators of triage, prognosis, and the extent of recovery in stroke patients. Hence, the present study addresses a striking gap in the literature to identify NMR-based urinary biomarkers for the longitudinal follow-up monitoring of stroke recovery that would be extremely valuable for clinical practice.

The present proof-of-principle study used ^1^H NMR metabolomic profiling combined with univariate statistics and multivariate machine learning to identify novel urinary biomarkers indicative of recovery processes and treatment responses in stroke. This study was designed to evaluate the metabolomic profiles in the acute phase (median = 5 days) following stroke and 6 months thereafter, with the aim of identifying the specific metabolites responsible for observed differences and the underlying biochemical pathways that contribute to symptoms and recovery. It was hypothesized that recovery after a stroke results in significant changes in urinary metabolic profiles that correlate with clinical measures of function and reveal involvement in recovery processes. The altered metabolites were expected to aid in determining injury severity, prognosis, and potential recovery while uncovering significant biological pathways involved in the repair and recovery process after stroke.

## 2. Materials and Methods

### 2.1. Stroke Patient Information and Urine Sample Collection

The present investigation was part of the project called Understanding Neurological Recovery: the role of resting state fMRI, biomarkers, and robotics after traumatic brain injury, stroke, and spinal cord injury (UCAN), aimed at following participants’ recovery after stroke up until around 6 months post-lesion. Participants with stroke were recruited from the inpatient stroke unit at the Foothills Medical Centre, which has a campus at the University of Calgary, where they were approached by their circle of care to participate in this study. After obtaining consent to contact, a researcher from the UCAN team provided the patients with detailed information about the study and obtained their informed consent. A total of 19 stroke participants were recruited for the study, of which 10 provided 2 urine samples, including 7 participants with ischemic stroke and 3 with hemorrhagic stroke (average age 61.5 ± 13.2 years). Fasting morning urine samples, acquired between 6:00 a.m. and 8:00 a.m., were collected at two time points: in the acute/early subacute phase after stroke within 2–11 days, hereafter referred to as the acute phase due to the median of the collection days (median = 5, interquartile range = 2), and again in the chronic phase 6 months post-injury, within 101–242 days (median = 203, interquartile range = 29.5). For sample collection, participants used an antiseptic alcohol wipe to clean the urethral opening and filled the collection cup approximately halfway with a midstream urine sample. Urine samples were stored at −80 °C and sent to the University of Lethbridge for further processing. Pairwise analyses were performed on subjects to best control for confounding variables such as diet, lifestyle, body mass index, medical history, and acute versus chronic drug treatments.

### 2.2. Clinical Measures

In this study, initial clinical assessments were taken within 4–32 days (median = 14.5, interquartile range = 12) and 6-month follow up assessments were completed 98–242 days (median = 203, interquartile range = 29.5) after stroke. The National Institutes of Health Stroke Scale (NIHSS) [26] was used at the initial time point to measure stroke severity using 15 items, including coordination, vision, speech, sensory, and motor symptoms. All items were recorded on a scale from 0 to 2, 0 to 3, or 0 to 4 and summed for a total possible score between 0 and 42, with a score of 42 indicating the most severe impairment. The Chedoke–McMaster Stroke Assessment Impairment Inventory (CMSA) [27] was also utilized to evaluate physical impairments and disabilities following stroke. The CMSA was conducted within a few days of injury and at 6-month follow-up, using a 7-point scale to measure arm and upper limb motor impairment, with 1 representing paralysis and 7 representing normal movement. This study focused on the hand and arm dimensions of the impairment inventory because the upper extremity is often found to be impaired post-stroke. Finally, The Functional Independence Measure (FIM) [28] was used to assess the level of disability in stroke patients based on motor control, self-care, communication, social skills, and cognitive skills. The FIM was administered both at baseline and at the 6-month follow-up. Functional scores on 18 items were recorded on a 7-point scale, with a total possible score of 126, indicating complete functional independence.

### 2.3. NMR Sample Preparation, Data Collection, and Post-Processing

Urine samples were removed from the −80 °C freezer and thawed at room temperature. To ensure a consistent pH and to minimize positional noise within the datasets generated by NMR analysis [29,30], urine samples were mixed with a buffer prepared in-house. The buffer consisted of dibasic potassium phosphate (K_2_HPO_4_) and monobasic potassium phosphate (KH_2_PO_4_) mixed in a ratio of 4:1 with a concentration of 0.625 M in deionized H_2_O. To inhibit microbial growth and to correct for the effect of divalent cations on chemical shift, 3.75 mM sodium azide (NaN_3_) and 0.375 mM potassium fluoride (KF), respectively, were added to the buffer. The buffer solution’s final pH was adjusted to 7.4 through the titration of either 3 M HCl or 3 M NaOH.

Each sample was prepared by adding 160 µL of buffer, 40 µL of D_2_O containing 0.02709% weight/volume trimethylsilyl propanoic acid (TSP), and 400 µL of urine. TSP was utilized as the chemical shift reference for this study. Samples were then mixed and pipetted into a 2 mL microfuge tube, centrifuged at 12,000 rpm for 5 min at 4 °C, and 550 µL of the supernatant was transferred to a 5 mm NMR tube for analysis. Using an NMR pH meter, the sample pH was checked to confirm that the sample complied with a pH of 7.4 ± 0.05.

A 700 MHz Bruker Avance III HD NMR spectrometer equipped with a room temperature triple resonance broad band observe (TBO) probe was utilized to acquire the NMR data. Prior to data acquisition, three-dimensional and one-dimensional shimming experiments were conducted to correct for any inhomogeneities in the static magnetic field. A one-dimensional nuclear overhauser effect spectroscopy (NOESY) experiment with gradient-based water suppression (pulse sequence ‘noesygppr1d’) with a mixing time of 10 ms was utilized to collect the NMR data [31]. The experiment was completed with 128 scans and 128 k data points. The spectra were pre-processed using Bruker Topspin software (version 3.2, patch level 6) by zero filling to 256 k points, line-broadening to 0.3 Hz, Fourier transforming, and referencing to the TSP peak (0.00δ). Additionally, automatic phase and baseline correction were applied to consistently enhance the quality of the spectra. Spectra were then exported as ASCII files to undergo further processing and statistical analysis in MATLAB.

Recursive segment-wise peak alignment [32] was applied to all spectra in MATLAB to correct for major and minor peak position variations in urine, which ensures accurate identification of metabolites and generation of valid results. Subsequently, dynamic adaptive binning was applied in MATLAB [33] and the bins underwent manual inspection and correction for errors. The regions corresponding to water and urea were removed from the bins to eliminate any interference from these highly variable regions.

### 2.4. Statistical Analysis

Using an exploratory untargeted approach, multivariate and univariate statistical analysis was utilized to assess whether the urinary metabolite profiles could distinguish between the acute and chronic phase samples. Prior to modeling, the data were normalized to the total metabolome (excluding water and urea), pareto-scaled, and log-transformed [34,35,36,37]. A total of 505 bins, corresponding to 505 NMR peaks and all possible metabolites detectable by NMR [8], were created and included in the statistical analysis.

Following this, we employed an ordinary least square (OLS) regression model, which is often used in clinical MRI studies prior to analysis [38], to remove the confounding effects of age and sex on the metabolomics data. This data pre-processing step used in NMR studies is precisely how the regression model was used in this study. The model was implemented in Python programming language using the widely used StatsModels package version 0.12.2, which is an open-source statistical software package (Python Software Foundation version 3.6.13, https://www.python.org/, accessed on 16 June 2021). Using the Anaconda distribution, packages including glob, numpy, pandas, and matplotlib were installed and used to generate an in-house script. Linear regression models, including simple and multiple linear regression, are commonly used in metabolomics to determine the relationship between metabolites and covariates or to compare metabolite concentrations across groups [39,40]. In addition, residuals calculated during linear regression can be used to regress out the effects of confounding factors as a data pre-processing step. The use of the OLS regression model allowed us to account for the variability in the data due to age and sex, thereby enhancing the accuracy and precision of our metabolomics analysis.

Both Variable Importance Analysis based on random Variable Combination (VIAVC) and univariate statistical tests were employed to analyze the data for significance. For univariate testing, either the paired *t*-test or paired Wilcoxon signed rank test were employed to analyze the parametric and non-parametric data, respectively. The Shapiro–Wilk test was used to determine if the data for each bin was parametric or not [41]. VIAVC is a machine learning algorithm implemented in MATLAB that identifies important metabolites in a dataset by using binary matrix resampling (BMR) to generate subsets of variables with equal probability [42]. These subsets are then used to perform Partial Least Squares-Linear Discriminant Analysis (PLS-LDA) with 10-fold (F-ranked variables) cross validation (CV) and double 10-fold (best subset) cross-validation (DCV), as well as the Receiver Operator Characteristic (ROC) test and subsequent Area-Under-the-Curve (AUC) analysis, to rank the importance of each metabolite [43,44]. Based on the metabolite’s interaction with other variables, VIAVC categorizes it as strongly informative, weakly informative, uninformative, or interfering. Through many iterations of removing uninformative and interfering variables, VIAVC outputs an F-ranked subset and a best subset of informative metabolites, both ranked by the Bonferroni–Holm-corrected *p*-value. Overall, VIAVC is a reliable technique to identify significant metabolites that lead to group classification due to its thorough approach to modeling and validation.

Both the univariate and multivariate statistical analysis were performed using the MetaboAnalystR version 2.0.4 package running inside R version 3.5.3 [45]. This included the univariate tests outlined above, as well OPLS-DA and ROC curve analysis. ROC curves were generated using the variables from the VIAVC best subset and utilized to visualize the sensitivity, specificity, and predictive accuracy of the biomarkers. OPLS-DA was carried out using the variables identified as significant by either the univariate or VIAVC tests.

Metabolites corresponding to significant bins were identified using a combination of resources, including Chenomx 8.2 NMR Suite, the Human Metabolome Database (HMBD) [46], and the Human Urine Metabolome paper [8]. Pathway topology analysis was performed using MetaboAnalyst selecting the Kyoto Encyclopedia of Genes and Genomes (KEGG) database for Homo sapiens as the pathway library, and the hypergeometric test was employed for over-representation analysis. Furthermore, relative-betweenness centrality was used for topology analysis [46,47]. Only the metabolites that were found to be significantly altered in this study were included in the pathway topology analysis.

To evaluate the relationship between the normalized urinary metabolite concentrations and the participants’ clinical assessment scores, Spearman rank-order correlations were calculated. Specifically, three sets of correlations were completed: first, between initial NIHSS, or CMSA, or FIM scores and acute metabolite concentrations; second, the percent difference of the participants’ CMSA or FIM scores and the acute concentrations; and third, the percent difference of the participants’ CMSA or FIM scores and percent difference of the normalized urinary metabolite concentrations. To control for multiple comparisons, Bonferroni correction was applied to adjust the significance threshold for our analysis. The Bonferroni-corrected *p*-value was obtained by dividing alpha < 0.05 by the number of VIAVC best subset bins tested in this study (*n*  =  8), giving a threshold of alpha = 0.00625. The percent differences were computed as follows:

(1) $$\frac{6\;months\;post\;injury-initial}{\left(\frac{6\;months\;post\;injury+initial}{2}\right)}\times 100$$

## 3. Results

### 3.1. Stroke Patient Characteristics

Table 1 presents clinical data collected in the acute and chronic phases for various clinical parameters and highlights differences in age, sex, stroke type, medications, and co-morbidities among participants. The table also summarizes information about the impact of stroke on impairments and functional independence, as evidenced by changes in NIHSS, FIM, CMSA-arm, and CMSA-hand scores. NIHSS scores (average 5.56 ± 3.64) show five stroke cases with mild stroke severity (NIHSS scores 4 or less) and four cases with moderate severity (NIHSS scores from 5 to 15). The majority of participants exhibited an improvement in clinical scores for FIM, CMSA-arm, and CMSA-hand, with an average improvement of 22.67 ± 14.13, 1.10 ± 1.29, and 0.70 ± 0.67, respectively. This improvement in clinical scores indicates recovery from stroke, with recovery being defined in this paper as the change in metabolite concentration and clinical parameters from initial collection to 6-months post-stroke.

### 3.2. Metabolomic Analysis of Urine Samples

To determine significant metabolic changes in urine, data analysis used VIAVC and paired *t*-test or Wilcoxon signed rank tests. The results of these analyses are presented in Table 2. Out of the 505 bins analyzed, 8 were significant according to VIAVC best subset analysis (corresponding to 8 different metabolites), 8 were significant via paired *t*-test or Wilcoxon signed rank (corresponding to 6 different metabolites), and 1 bin was significant based on both tests (13 total significant metabolites). The top three metabolites based on univariate analysis were pseudouridine, phenylacetic acid, and acetylcholine. Similarly, the top three metabolites in the VIAVC best subset were pseudouridine, 4-hydroxy-3-methoxymandelate, and inosine. Lastly, the bin that was found to be significant by both tests was identified as pseudouridine.

**Figure 1:**
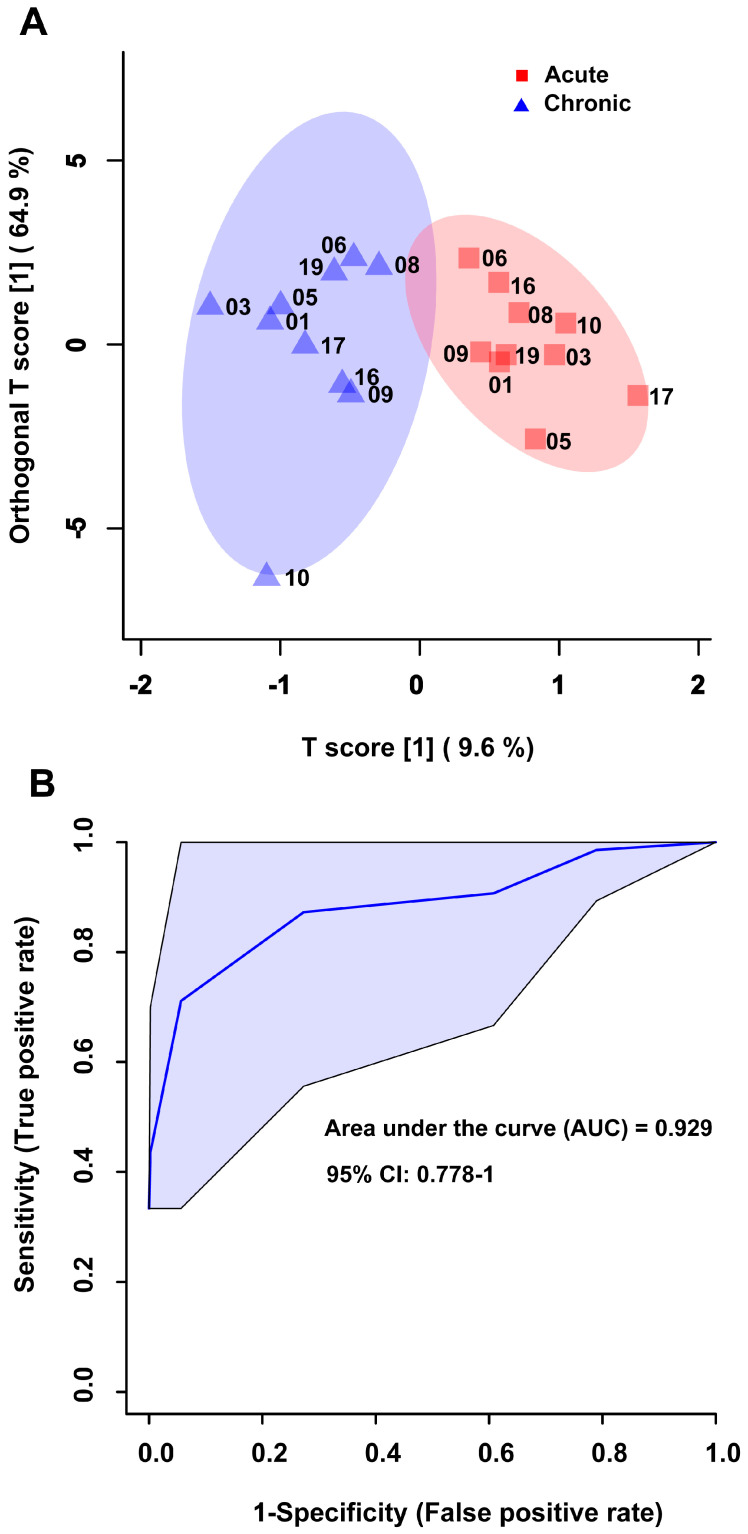
Orthogonal projections to latent structures discriminant analysis (OPLS-DA) scores plot and receiver operator characteristic (ROC) curve for the comparison of the acute stroke (red squares) and chronic stroke (indigo triangles) urine samples. The OPLS-DA scores plot (**A**) illustrates supervised group separation between the two groups with a Q^2^ = 0.775 (*p* < 5 × 10^−4^) and R^2^ = 0.836 (*p* < 5 × 10^−4^). The x-axis and y-axis of the scores plot represents the predictive variation between groups and the orthogonal variation within groups, respectively. The ROC curve (**B**) displays a high level of sensitivity and specificity when classifying the two groups with an area under the curve (AUC) of 0.929, a 95% confidence interval of 0.778–1, and a predictive accuracy of 85%. Both figures were created using the metabolites identified as significant by the VIAVC best subset analysis (8 of the 505 bins, see Table 2).

OPLS-DA scores plots reveal excellent group separation when comparing urine samples for acute to chronic stroke, as evidenced by little to no overlap of the confidence internals for the two groups, no sample misclassifications, and a Q^2^ value of 0.775 (Figure 1A). In addition, the ROC analysis illustrates the high sensitivity, specificity, and robustness of the model, with an AUC of 0.929, a 95% confidence interval of 0.778–1, and a predictive accuracy of 85% (Figure 1B). Both the OPLS-DA and ROC analyses were performed using the eight metabolites identified as significant by the VIAVC best subset testing (Table 2).

**Figure 2:**
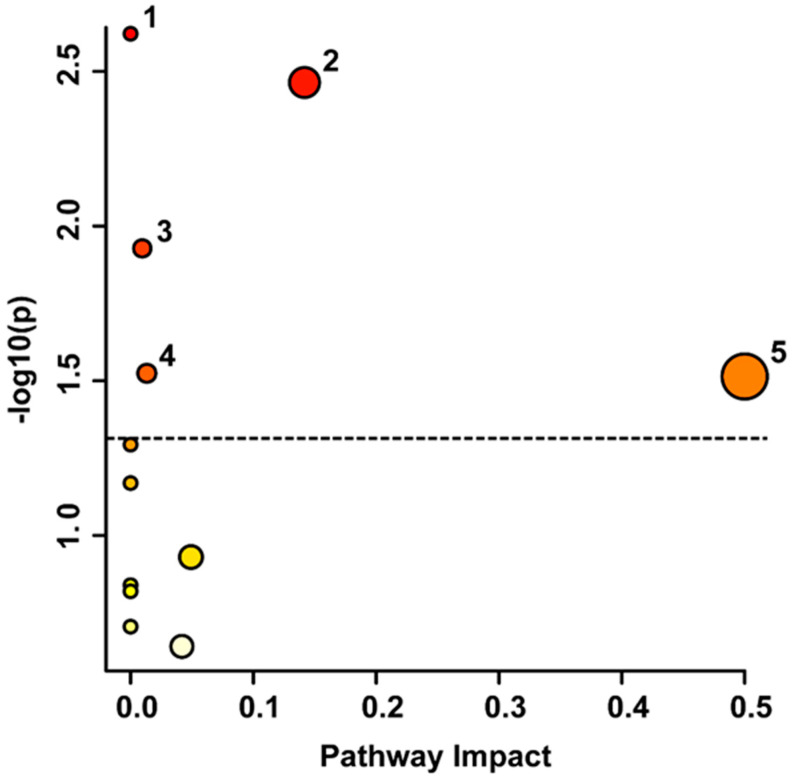
Metabolomic pathway topology analysis for the comparison of acute and chronic stroke generated using the list of metabolites identified as significant by VIAVC best subset analysis or a paired *t*-test (Table 1). The figure displays a visualization of metabolic pathways, with the y-axis indicating *p*-values for each pathway, and the x-axis representing the pathway impact, which reflects the extent significant metabolites are able to alter pathway function. The circles represent each pathway, with circle colour indicating degree of significance, and circle size indicating pathway impact. The labelled pathways with *p* < 0.05 (significance threshold indicated by the dotted line) and highest pathway impact are highlighted and numbered as follows: 1. Phenylalanine Metabolism (*p* = 0.002), 2. Tyrosine Metabolism (*p* = 0.003), 3. Purine Metabolism (*p* = 0.011), 4. Glycerophospholipid Metabolism (*p* = 0.030), and 5. Phenylalanine, Tyrosine, and Tryptophan Biosynthesis (*p* = 0.031).

The urinary metabolites identified as significant based on a VIAVC best subset or paired *t*-test were potentially involved in several pathways, as indicated by Figure 2. Notably, phenylalanine metabolism (*p* = 0.002) displayed the highest significance and the metabolites within the phenylalanine, tyrosine, and tryptophan biosynthesis (*p* = 0.031) pathway resulted in the highest pathway impact. The tyrosine metabolism (*p* = 0.003), purine metabolism (*p* = 0.011), and glycerophospholipid metabolism (*p* = 0.030) pathways were also found to be significantly altered from acute stroke to chronic stroke. Pathway analysis is used to provide insight into the metabolic changes occurring after stroke and highlight potential targets for future therapeutic interventions or biomarkers for stroke diagnosis and treatment.

### 3.3. Correlation of Metabolomic Signatures Linked to Motor Recovery

**Figure 3:**
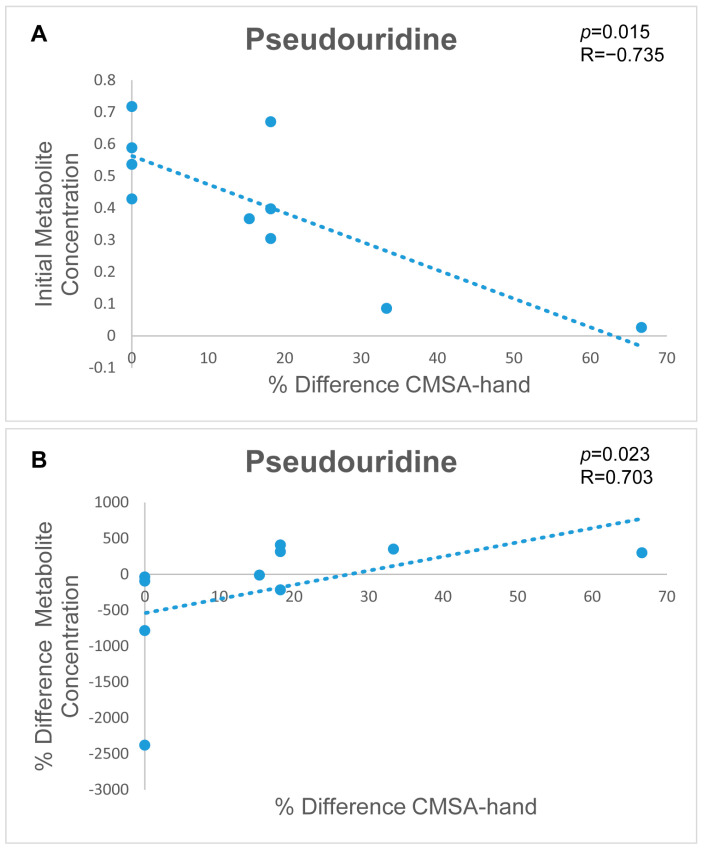
Scatterplots for visualization of significant Spearman correlations and corresponding correlation coefficients (R) and *p*-values for two distinct correlation analyses: (**A**) acute pseudouridine concentration correlated to the percent difference in the Chedoke–McMaster Stroke Assessment of the hand (CMSA-hand) scores and (**B**) the percent difference in pseudouridine concentration correlated to the percent difference in the CMSA-hand scores. The x-axis illustrates the percent difference in CMSA-hand scores, while the y-axis provides (**A**) the acute pseudouridine concentration or (**B**) the percent difference in pseudouridine concentration. For the first comparison, a negative relationship indicates that lower levels of pseudouridine correlate to a greater percent difference, or improvement, in CMSA-hand scores. For the second comparison, a positive relationship reveals that a greater increase in metabolite concentration from the acute time point to the chronic time point correlates to a larger percent difference, or improvement in CMSA-hand scores.

Spearman correlations between significant metabolites and the CMSA-hand scores were conducted and the significant results are visualized in Figure 3. Pseudouridine, the urinary metabolite with the topmost VIAVC best subset and univariate significance, illustrated a strong correlation to the CMSA-hand scores in two separate correlation analyses: the acute metabolite concentration correlated to the percent difference in CMSA-hand scores, and the percent difference in metabolite concentration correlated to the percent difference in CMSA-hand scores. The former displays a positive correlation and the latter yields a negative correlation. None of the correlations passed the Bonferroni-corrected threshold of alpha = 0.00625. The significant Spearman correlation values for pseudouridine and the CMSA-hand scores suggest a potential relationship between the urinary metabolite and motor recovery following stroke.

## 4. Discussion

This study pursued a characterization of metabolites and their associated metabolic pathways linked to stroke over time, revealing a notable relationship between pseudouridine and motor recovery. Specifically, the differences in the metabolic profiles were evident when comparing acute stroke samples to those 6 months later, five significantly altered metabolic pathways revealed the pathophysiology underlying stroke, and clinical correlations identified pseudouridine as a potential biomarker of change over time. The present findings provide new avenues for understanding the underlying biological mechanisms that contribute to post-stroke recovery and may lead to the development of targeted therapeutic interventions and the improvement of the clinical management of stroke patients. In the following discussion, we will examine these results in greater detail and discuss their implications for future research and clinical practice.

### 4.1. Clinical Translation and Classification of Stroke Metabolites

The OPLS-DA scores plot provided an effective means for visualizing the separation of the supervised groups and the significance of the metabolite subsets in predicting post-stroke outcomes. The OPLS-DA scores plot seen in Figure 1A illustrates a significant difference in the metabolomic profiles of individuals initially after stroke compared with those at 6 months post-stroke, and that these differences can be detected using multivariate statistical analysis. Cross validation and permutation measures indicated that the model have robust predictive ability, with the VIAVC best subset metabolites providing the best classification. The ROC and corresponding AUC (Figure 1B) highlight the diagnostic accuracy of the VIAVC best subset of metabolites for distinguishing between acute and chronic stroke samples. An AUC of 1.0 represents perfect classification, while an AUC of 0.5 represents random guessing and an AUC of less than 0.5 represents worse than random classification [42]. The ROC curve has an AUC of 0.929, with a 95% confidence interval of 0.778–1, indicating the strong predictive ability of the model and that the results are statistically significant. The predictive accuracy of the model of 85% indicates that the metabolite panel can correctly classify 85% of samples into their respective groups. These findings suggest that the metabolites identified through the VIAVC best subset analysis can serve as potential biomarkers for effectively distinguishing between acute and chronic stroke samples.

### 4.2. Metabolic Pathways Involved in the Stroke Recovery Process

#### 4.2.1. Phenylalanine Pathways

Phenylalanine metabolism was significantly altered between the acute and chronic phases of stroke. Previous studies have indicated that phenylalanine metabolism is upregulated in the acute phase after stroke [25]. Feeding into this metabolic pathway and the tyrosine metabolism pathway is the phenylalanine, tyrosine, and tryptophan biosynthesis pathway, which was also significantly altered. Previous studies have indicated that phenylalanine, tyrosine, and tryptophan biosynthesis is significantly affected in acute ischemic stroke [48]. Accumulating evidence links both of these phenylalanine pathways to alterations in post-stroke depression [49,50]. Phenylalanine exhibits multiple metabolic pathways within the body, including its incorporation into proteins, conversion into phenylpyruvic acid, and conversion into tyrosine. High levels of phenylalanine and its derivatives, including phenylacetic acid, exacerbates oxidative stress, elicits lipid peroxidation [51], and impairs synaptogenesis in rodents and humans [52]. Oxidative stress is a critical contributing factor to stroke pathology and phenylalanine may play a role in its progression and recovery. Interestingly, during lipid peroxidation, reactive oxygen species (ROS) attack and cause damage to glycerophospholipids. Furthermore, lipid peroxidation can have a cascading effect when its products interact with proteins, deoxyribonucleic acid (DNA), and other lipids, leading to additional oxidative damage and the disruption of cellular functions. Even alone, phenylalanine and its metabolites can damage DNA by attacking purine and pyrimidine bases and deoxyribose sugar [53]. Damage to glycerophopholipids and DNA alterations are further discussed below in Section 4.2.4. It is imperative to consider that the discussed studies focused on high levels of phenylalanine akin to those in phenylketonuria, and different phenylalanine levels may yield different effects. Regardless, the significant alteration in phenylalanine metabolism, its metabolites, and its association with the biosynthesis pathway highlight the potential role of phenylalanine in stroke progression.

#### 4.2.2. Tyrosine Metabolism

The present stroke study revealed that tyrosine metabolism was potentially disturbed. Prior research corroborates this finding, showing that tyrosine metabolism is impacted during cerebral infarction [54]. Tyrosine is the precursor for catecholamine synthesis [55] and is significantly downregulated over the course of this study. The downregulation of tyrosine could lead to the decreased production of catecholamines, leading to lower levels of tyrosine being used for their synthesis. These results, which have been corroborated by previous research, illustrate that during stress or stroke, more tyrosine is needed and catecholamines become depleted [56,57]. 4-hydroxy-3-methoxymandelate, otherwise known as vanillylmandelic acid (VMA), and homovanillate or homovanillic acid (HVA) are both end-products of catecholamine metabolism [58]. The upregulation of VMA and HVA in urine after stroke observed in this study may indicate the increased activation of the central nervous system leading to the increased breakdown of catecholamines and these metabolites have previously been identified as prognostic biomarkers for stroke [24]. The metabolite tyrosine is frequently found to be significantly altered in stroke [25,56] and post-stroke depression [49,50,59]. While Ormstad et al. [56] argue that tyrosine levels decrease after stroke, other findings suggest that the concentration of tyrosine is increased in the acute phase [25]. This discrepancy may derive from differences in methodology or sample-collection times. More research is needed to decipher the complexity of the tyrosine and catecholamine response after stroke.

#### 4.2.3. Purine Metabolism

Purine metabolism is another pathway that was changed after stroke. Earlier investigations implicated purine metabolism in ischemic stroke [60,61,62]. For instance, purine metabolites, such as inosine and adenosine, have proven to be useful in determining stroke severity irrespective of stroke type [63]. Researchers have proposed that the neurotoxic effects of oxidative stress and inflammation from stroke are offset by intermediates of purine metabolism [60,64]. Purines such as adenosine 5′-triphosphate (ATP), adenosine, and inosine are rapidly released following injury such as stroke to minimize damage and support healing [64,65] and during these hypoxic conditions, ATP is metabolized into adenosine, and further, inosine [66]. Microglial cells are recruited to the injury site where they are activated by purines to aid repair by releasing inflammatory cytokines [67]. Adenosine is a purine nucleoside that is neuroprotective after stroke owing to its numerous abilities, including controlling blood flow, maintaining homeostasis, regulating the immune response, reducing respiration, preventing the breakdown of triacylglycerols, and inhibiting the production of neurotoxic pro-inflammatory cytokines [68,69,70,71]. Similarly, inosine, a modified adenosine in RNA, has also displayed anti-inflammatory and neuroprotective effects after stroke [72]. Inosine has shown potential in neural repair after CNS injury [73] and stroke through its promotion of axon growth and alterations in gene expression [74,75]. Moreover, ATP is released after injury as a co-transmitter with acetylcholine [76] and catecholamines [77], and both of these neurotransmitters and their associated metabolites were also significantly altered and are discussed in this section. It is important to note that adenosine and inosine are involved in a wide variety of cellular processes, such as being precursors to DNA and ribonucleic acid (RNA) as previously discussed. These metabolites may simultaneously be used to synthesize and repair damaged DNA caused by the brain injury. Taking all of this into consideration, the purines are diverse metabolites that play numerous roles in the pathophysiological response to brain injury.

#### 4.2.4. Glycerophospholipid Metabolism

Glycerophospholipid metabolism is another possible pathway that is perturbed following stroke, as confirmed by other studies investigating stroke [48,60,61,78,79,80] and post-stroke depression [81]. Glycerophospholipids play diverse roles in neural membranes, providing structure, maintaining homeostasis, and facilitating ion permeability. Upon injury, membrane glycerophospholipids may become damaged, and the subsequent neuroinflammatory response can lead to the breakdown of glycerophospholipids into lipid mediators that regulate neuroinflammation, oxidative stress, neural cell proliferation, differentiation, and apoptosis [82]. Ethanolamine is a precursor for acetylcholine [83] and phospholipids and previous research supports the findings of this study that demonstrate ethanolamine is increased following stroke [84]. Studies have shown that dendrite growth is dependent on the glycerophospholipid phosphatidylethanolamine, which is derived from ethanolamine [85]. Since neural expansion and reformation is needed after the damage caused by stroke, this could potentially be a mechanism to promote recovery. Further, compelling evidence has indicated that the α-7 nicotinic acetylcholine receptor (α-7 nAChR) plays a role in neuroprotection by reducing neuroinflammation and oxidative stress in stroke [86]. The activation of the α-7 nAChR by acetylcholine or other agonists has beneficial effects in experimental models of stroke through the activation of downstream signalling mechanisms that can mitigate the damaging effects of stroke. Given that acetylcholine activates many other receptors and has a complex impact on the brain, further research is needed to fully understand the neuroprotective role of acetylcholine and its potential therapeutic applications in stroke. All encompassing, the perturbation of glycerophospholipid metabolism is evident following stroke and the potential neuroprotective role of ethanolamine and acetylcholine warrant further investigation to deepen the understanding of their therapeutic applications in stroke.

### 4.3. The Relationship of Metabolic Biomarkers to Clinical Parameters

#### 4.3.1. Metabolic Signatures to Predict Stroke Patient Outcomes

Pseudouridine is a modified nucleoside that is a component of RNA and its metabolite is associated with RNA stability and translation [87]. Post-transcriptionally regulating RNA expression involves modifications after transcription and synthesis. Among these modifications, pseudouridine stands out as the most prevalent post-transcriptional alteration [88]. Decreased levels of pseudouridine in urine may suggest impaired RNA synthesis or degradation, which may affect the cells’ ability to respond to injury and recovery. Pseudouridine has been reliably established as a biomarker for post-stroke depression [89] and ischemic stroke, even after accounting for risk factors [90]. In this study, the urinary metabolite pseudouridine showcases potential in prognosticating stroke patient outcomes through a significant Spearman correlation (R = −0.735, *p* = 0.015). The pseudouridine concentration during acute stroke injury was correlated to the CMSA-hand score percent difference. This correlation resulted in a negative relationship, indicating that decreased levels of urinary pseudouridine were linked with greater improvements in CMSA-hand scores. This preliminary finding illustrates that pseudouridine holds promise as a prognostic indicator for stroke, with lower levels correlating with improved stroke patient outcomes.

#### 4.3.2. Metabolic Signatures to Monitor Stroke Patient Recovery

Pseudouridine showed a significant correlation (R = 0.703, *p* = 0.023) between the percent difference in pseudouridine concentration of the acute to chronic sample collections and the percent difference in the CMSA-hand scores, taken at the same time-points. In the previous section, the involvement of pseudouridine in RNA synthesis and degradation was outlined, along with its association with stroke [90]. The present positive correlation suggests that an increase in pseudouridine concentration during the chronic phase when compared with the concentration during the acute phase is associated with a significant improvement in CMSA-hand scores. Thus, monitoring pseudouridine levels shows potential for stroke prognosis. These findings highlight the clinical significance of pseudouridines potential as a valuable marker for assessing therapeutic effectiveness and predicting functional recovery following stroke.

There are several limitations with this research that must be addressed. First, the limited sample size and diversity in confounding factors such as differences in diet, exercise, mobility, body mass index, injury level, acute versus chronic drug treatment, and medical history need to be considered. In addition to this, a more diverse sample group with an equal number of females would be beneficial to this research. Another limitation in the present investigation is the lack of a baseline or control group such as a musculoskeletal injury group that allows for a comparison to pathophysiological changes after injury in general. In the absence of a musculoskeletal injury control group, discerning alterations specifically attributed to stroke from those stemming from general injury becomes challenging. For studies that use urine as the primary biofluid similarly to this study, the evaluation of renal function, such as glomerular filtration rate and tubular function, would be beneficial. Further, there is a large amount of heterogeneity within our stroke sample, and it is important to note that different stroke types and locations may impact biomarkers. Future studies would benefit from taking these factors into consideration when developing a study design. The present study collected initial urine samples during the acute phase, with a couple of samples going into the early subacute phase. Future investigations would benefit from strict sample collection cut-offs that follow the international guidelines for critical time-points during stroke recovery [91]. The longitudinal design of this study helps mitigate many of these confounds by pairwise analysis of the samples, ensuring participants are being compared with themselves only. Moreover, the linear regression model accounted for variations in age and sex to guarantee that these variables did not sully the results. Regardless, the present results have important implications for stroke research and treatment and warrant further investigation into the role of urinary metabolites in post-stroke metabolic changes and recovery. Overall, this foundational research sheds light on the metabolomic changes associated with stroke and their potential role in the pathophysiology of stroke, as well as the potential of these metabolites as biomarkers for stroke diagnosis and treatment.

## 5. Conclusions

In conclusion, this pilot study provides valuable insights into the pathophysiological changes that occur following stroke and proposes pseudouridine as a marker for stroke prognosis and treatment monitoring. Specifically, the results indicate that metabolomic analysis can identify specific metabolites and pathways that are significantly altered following a stroke, thereby shedding light on the underlying biological mechanisms involved in post-stroke recovery. This study identified a panel of metabolites that showed high sensitivity and specificity in distinguishing between acute stroke and chronic stroke in urine, indicating their potential as biomarkers for stroke diagnosis. Furthermore, this study revealed significant correlations between urinary pseudouridine levels and CMSA-hand scores, thereby emphasizing pseudouridine as a potential valuable tool in stroke management.

The preliminary findings highlight the importance of metabolic pathways in stroke recovery processes. This study identified significant alterations in phenylalanine metabolism, tyrosine metabolism, purine metabolism, glycerophospholipid metabolism, and phenylalanine, tyrosine, and tryptophan biosynthesis. These pathways are involved in various physiological processes, such as gene expression, neurotransmitter synthesis and metabolism, oxidative stress, and neuroinflammation. The dysregulation of these pathways may contribute to the pathophysiology of stroke and provide potential targets for therapeutic interventions. The significance of this study stems from the capacity of NMR-driven metabolomic biomarkers to transform into cost-effective, rapid assays for biomarker detection that could find utility in a pre-hospital setting [92].

Moreover, this exploratory investigation provides initial evidence for the reliability and clinical translation of urine-based metabolomic signatures as biomarkers for stroke. The OPLS-DA scores plot illustrated the ability to distinguish between acute and chronic stroke samples using multivariate statistical analysis. Furthermore, the VIAVC metabolite panel showed a high level of diagnostic accuracy, sensitivity, and specificity in classifying the samples. In addition, the urinary metabolite pseudouridine has potential clinical importance as a biomarker prognosticating stroke outcomes and monitoring treatment success. These findings have implications for improving stroke diagnosis and clinical management, as well as for the development of targeted therapeutic interventions.

Overall, this comprehensive analysis of metabolomic changes in stroke patients contributes to the understanding of post-stroke recovery mechanisms and opens new avenues for future research and clinical practice. The identified metabolites and pathways provide potential targets for therapeutic interventions and biomarker development, ultimately aiming to improve the outcomes and quality of life for stroke patients. Further research is needed to validate these findings and explore their applicability in larger patient populations.

## Figures and Tables

**Table 1:** Clinical and demographic characteristics of *n* = 10 stroke patients indicating the stroke type, vascular territory, side of the brain affected by stroke, the days between stroke, and both acute and chronic sample collection, age, sex, medications, co-morbidities, and various stroke scale scores. These clinical assessments include initial NIHSS scores along with initial and 6-month FIM, CMSA-arm, and CMSA-hand scores.

Patient Code	Stroke Type	Vascular Territory	Affected Side	Sex	Age	Urine Collection (Days Post-Stroke)		Medications	Co-Morbidities		NIHSS	**FIM**	**CMSA-Hand**		**CMSA-Arm**	
						Initial	6 Month				Initial	**6 Month**	**Initial**	**6 Month**	**Initial**	**6 Month**
ST-01	Ischemic	MCA	Left	M	79	2	242	ASA, Atorvastatin, Clopidogrel, Docusate Sodium, Perindopril	A Fib, acute renal failure, motor-cycle accident 1952—Knocked out; right collar bone fracture, Left leg injury d/t combine accident as a teen	NaN	109	123	5	6	5	7
ST-03	Ischemic	LACUNAR	Right	M	37	5	221	ASA, Synthroid, Rosuvastatin, HCTZ, Felodipine	Hypothyriodism, HTN, smoker (1/2 ppd × 5 years), EtOH	11	92	120	1	2	1	3
ST-05	Ischemic	MCA	Left	M	47	6	206	ASA, Crestor	HTN, smoker (30 per day)	1	116	125	5	7	7	7
ST-06	Ischemic	MCA	Left	M	64	4	101	Warfarin, Solatol, Metoprolol, Atorvastatin, Levothyroxine, Vit D, Calcium, Magnesium, Benzaclin Pump	A Fib, valve disease—dilated cardiac myopathy 1990, irregular heart rate in 2002, 2016, cardiac MRI performed, no clot found.	3	105	115	5	5	6	5
ST-08	Ischemic	MCA/ACA	Left	M	62	4	200		Diabetes, smoker, increased cholesterol	2	106	124	6	7	5	7
ST-09	HEM	MCA	Left	F	61	5	191	Gravol, Gabapentin, Lovenox, Lantus, Norvasc, Pantoprazole, Restorlax, Aidactone, Vit D, Ativan, Advair	HTN, diabetes, asthma, Barrett’s esophagus, obstructive sleep apnea, chronic neck and low back pain—recurring PRP treatment	9	80	124	5	6	4	7
ST-10	Ischemic	Thalamus	Left	M	72	6	189	ASA, Diamicron, Metoprolol, Fosinopril, Gliclazide, Plavix, Metformin, Atorvastatin, Vit D, Lantus, Humulin, Drug Study (Rivanrobran vs Placebo)	HTN, diabetes, hyperlipidemia, ischemic heart disease, CABG (Aug 2015), post-CABG enrolled in COMPESS, TIA on 11 January 2017 sent to stroke prevention clinic on 13 January 2017 and then admitted to acute stroke unit	4	96	122	5	6	4	6
ST-16	HEM	MCA	Left	M	62	8	213	Nicotine, Amlodipine, Baclofen, Acetominaphen	HTN, hyperlipidemia, smoker, prostate cancer, degenerative changes spine, chronic sinusitis	10	70	115	5	5	4	5
ST-17	Ischemic	MCA + ICA	Left	M	53	11	221			6	NaN	126	6	6	6	6
ST-19	HEM	PCA	Left	F	78	4	170	Amlodipine, Enoxaparin, Hydrochlorothiazide, Nitro patch Polythylene Glycol	HTN, hyperlipidemia, cleft Palate	4	113	123	7	7	7	7

Abbreviations: HEM = hemorrhagic, MCA = middle cerebral artery, ACA = anterior cerebral artery, PCA = posterior cerebral artery, ICA = internal carotid artery, ASA = acetylsalicylic acid, HCTZ = Hydrochlorothiazide, PRP = platelet-rich plasma, CABG = coronary artery bypass graft, HTN = hypertension, TIA = transient ischemic attack.

**Table 2:** Comprehensive summary of the significant urinary stroke metabolites arranged in descending order of VIAVC significance, followed by significance of the paired *t* or Wilcoxon signed rank test. The metabolites are identified by chemical shift in parts per million (ppm) along with their corresponding regulation. Metabolites with more than one resonance peak identified as significant are denoted as metabolite.1, metabolite.2, … metabolite.n.

Metabolite	Chemical Shift (ppm)	VIAVC *p*-Value	Paired t/Wilcoxon *p*-Value	Regulation
Pseudouridine.1	4.299	4.55 × 10^−23^	0.0020 (W)	Down
4-Hydroxy-3-Methoxymandelate	3.887	8.19 × 10^−19^	0.3567	Up
Inosine	8.219	1.59 × 10^−16^	0.1496	Down
Homovanillate	3.874	7.57 × 10^−16^	0.5282	Up
Adenosine	4.315	7.82 × 10^−16^	0.269	Down
2-Aminobutyrate	0.982	1.27 × 10^−15^	0.3256	Down
Ethanolamine	3.156	5.08 × 10^−15^	0.7229	Up
Deoxyinosine	6.494	3.41 × 10^−13^	0.6182	Up
Phenylacetic acid.1	7.328	Not Sig.	0.0124	Down
Phenylacetic acid.2	7.312	Not Sig.	0.0132	Down
Acetylcholine	2.157	Not Sig.	0.021	Down
L-Tyrosine	6.907	Not Sig.	0.0281	Down
Anserine	7.138	Not Sig.	0.0362	Down
Pseudouridine.2	4.29	Not Sig.	0.0137 (W)	Down
Alanine	1.493	Not Sig.	0.0371 (W)	Up

## Data Availability

The data featured in this study can be obtained by reaching out to the corresponding authors directly, as they have not been made accessible via an online database.
